# Promising Application of D-Amino Acids toward Clinical Therapy

**DOI:** 10.3390/ijms231810794

**Published:** 2022-09-16

**Authors:** Yoahpoing Shi, Zahid Hussain, Yufen Zhao

**Affiliations:** 1 Institute of Drug Discovery Technology, Ningbo University, Ningbo 315000, China; 2 Qian Xuesen Collaborative Research Center of Astrochemistry and Space Life Sciences, Ningbo University, Ningbo 315000, China

**Keywords:** D-amino acid, therapeutic effect, protective effect

## Abstract

The versatile roles of D-amino acids (D-AAs) in foods, diseases, and organisms, etc., have been widely reported. They have been regarded, not only as biomarkers of diseases but also as regulators of the physiological function of organisms. Over the past few decades, increasing data has revealed that D-AAs have great potential in treating disease. D-AAs also showed overwhelming success in disengaging biofilm, which might provide promise to inhibit microbial infection. Moreover, it can effectively restrain the growth of cancer cells. Herein, we reviewed recent reports on the potential of D-AAs as therapeutic agents for treating neurological disease or tissue/organ injury, ameliorating reproduction function, preventing biofilm infection, and inhibiting cancer cell growth. Additionally, we also reviewed the potential application of D-AAs in drug modification, such as improving biostability and efficiency, which has a better effect on therapy or diagnosis.

## 1. Introduction

**Figure 1:**
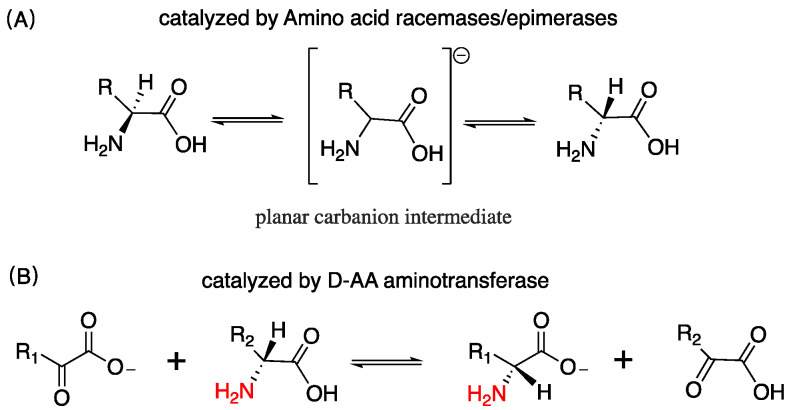
D-AAs are transformed or assembled by varied enzymes, both of them are reversible; (**A**) racemization via a planar carbanion intermediate; (**B**) the amino of one of D-AA is transferred to an α-ketoacid by D-AA aminotransferase, and generating the corresponding D-AA.

D-AAs are ubiquitous in nature, exist in bacteria, plants, and mammals, etc. [1,2,3]. In the exploration of the origin of life, the Miller–Urey experiment reveals the most possible synthetic pathway of D/L-AAs in nature [4]. Heating, acid and alkali treatments, etc. can be another source of D-AAs in the abiotic environment that convert L-AAs into corresponding mirror structures [5]. Organisms can endogenously produce D/L-AAs. Bacteria can synthesize D-AAs through racemization induced by AA racemase or epimerase, as well as catalysis induced by D-AAs aminotransferase enzymes, which utilize an amino donor substrate and an α-ketoacid substrate to assemble D-AAs, as shown in Figure 1 [6,7]. D-AAs exist widely in microbes; it governs cell wall remodeling in bacteria [8]. Particularly, D-Ala and D-Glu are highly prevalent among bacterial peptidoglycan [9]. In plants, D-AAs are adopted as a nitrogen source, building blocks, and signaling molecules [10]. It accounts for approximately 1.5% in the total AA pool of different plant parts [11]. In animals and human, D-Ser, D-Ala, and D-Asp have been found to be the most abundant D-AAs in neuroendocrine and endocrine tissues [12]. In the rat frontal brain area, the content of D-Ser is more than 200 nmol/g wet tissue [13]. D-Asp in some rat pituitary gland was detected as over 3000 nmol/g wet tissue [12], and in the rat pancreas, the amount of D-Ala can reach up to 450 nmol/g wet tissue [12]. Additionally, D-AAs containing peptides and protein have been discovered in organisms [14,15]. 

**Figure 2:**
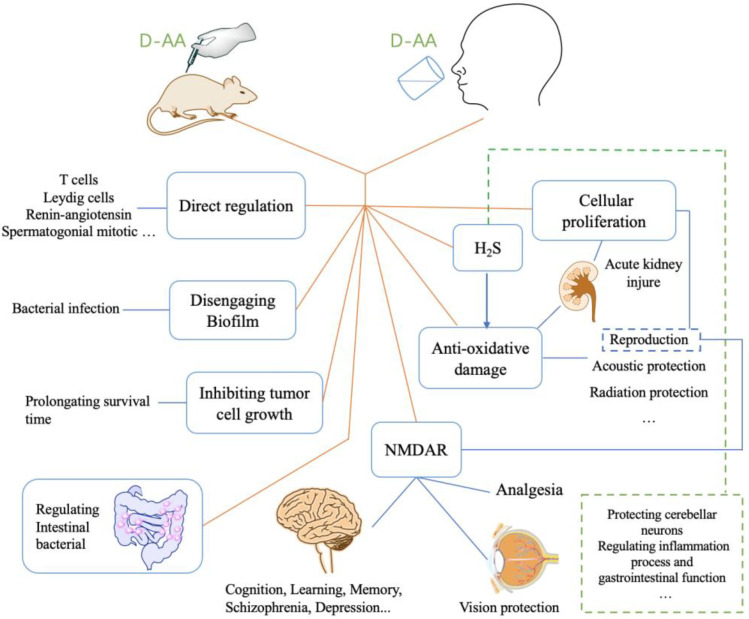
The relevant diseases/symptoms improved by D-AAs administration were through its direct and indirect action. NMDAR pathway is vital to ameliorate many symptoms with respect to psychological and physiological defects, such as depression, and vision, etc. H_2_S can be generated from the metabolic process of D-AA. H_2_S is important to gastrointestinal function, and inflammation process, etc. D-AAs have both direct and indirect action on anti-oxidative damage, whereas some acoustic protection, and radiation protection, etc. are associated with anti-oxidative damage. Inhibiting tumor cell growth, disengaging biofilm, regulating intestinal bacterial, and regulating T cells, etc. are important elements of the protective effect of D-AAs.

D-AAs have different physiological behaviors and activities in organisms compared to its L enantiomers, and it plays a vital role in life activity [1]. It has been found that it involves in lots of pathological processes, including chronic kidney disease, gastric cancer, psychosis, Alzheimer’s Disease (AD), etc. [16,17,18,19]. In some diseases, the levels of relevant D-AAs were lower than normal control [19]. Therefore, increasing the levels of D-AAs in organisms has been put forward for therapy. The strategy of inhibiting D-Amino acid oxidase (DAAO) was used for elevating the levels of D-AAs and has been proven feasible [20]. Another strategy, the injection or oral administration of D-AAs, was also suggested to be successful [19]. In the past few decades, numerous findings revealed that D-AAs effectively attenuate the symptom of disease or offer pre-protection from the coming disease (Figure 2). Here, we summarized the therapeutic function of D-AAs, as well as its potential application in future therapy.

## 2. The Treatment of Diseases Relating to Nervous System with D-AAs

### 2.1. Schizophrenia and Post-Traumatic Stress Disorder

N-Methyl-D-Aspartate receptors (NMDARs) are ionotropic glutamate-gated receptors involving synaptic plasticity, learning, memory, and pathological processes, etc., which consist of several homologous subunits of GluN1-, GluN2-, or GluN3-type [21,22]. D-Serine is an endogenous and potent co-agonist of NMDAR, which distributes in the hypothalamic structure and especially in the forebrain structure [23,24,25,26]. D-Ser and D-Ala are recognized as the most effective co-agonist of NMDAR among several analogs of glycine [27]. Moreover, D-Ser is up to three times more potent than glycine at the glycine site of NMDAR [28]. Earlier clinical trials of administrating placebo and NMDA antagonists gave rise to similar symptoms with schizophrenia, implying it might be derived from hypofunction of the NMDAR [27,29]. To date, more and more research has confirmed the relationship between NMDAR and various psychiatric conditions [30,31,32]. Activating NMDAR is therefore a new pharmacological pathway [33,34]. In schizophrenia patients, the level of D-Ser was decreased by 25% in cerebrospinal fluid, whilst the frontal cortex and hippocampal serine racemase protein levels were significantly reduced by 39% and 21%, respectively [35]. 

Tsai et al. have pioneering work on the treatment of schizophrenia with D-AAs, they corroborated that D-Ala or D-Ser can serve as a therapeutic agent [19,36]. In the case of D-Ser, the symptoms of schizophrenia were improved at week 2 and became more remarkable at week 4 and 6 though orally administrating D-Ser 30 mg/kg daily. At the end of the 6-week trial, a 17% reduction had occurred in the positive symptoms and a 21% reduction had occurred in the negative symptoms [19]. From admission to discharge, the level of D-Ser was prominently increased in schizophrenic patients [37]. In another trial, the D-Ala treatment showed a 17% reduction in the negative symptoms and a 13% reduction in the positive symptoms over a 6-week trial of orally administrating D-Ala 100 mg/kg/day [36]. Notably, the effective therapeutic dose of D-Ser was lower than that of D-Ala.

Post-traumatic stress disorder (PTSD) is a sort of psychosis relating to physiological response to traumatic events; the behaviors include emotional numbing, hyperarousal, and avoidance of stimuli, etc. [38]. D-Cycloserine had shown success in the treatment of PTSD before [39]. It was later found that D-Ser is also effective for the treatment of PTSD through enhancing the function of NMDAR in the clinical trial, and D-Ser has no significant side effects [40]. The reduction in symptom severity was observed at 6 weeks’ treatment via orally administrating D-Ser 30 mg/kg/day [40].

### 2.2. Parkinson’s Disease and Seizure

The symptoms of Parkinson’s Disease (PD) (rigidity, hypokinesia, and loss of postural balance, etc.) were significantly improved by D-Ser treatment over six consecutive weeks [41]. It may have been through activating NMDAR [41]. D-Ser is also effective in preventing temporal lobe epileptogenesis (TLE) as well as reducing the severity of seizures [42,43]. The relevant investigation probed that intracranially injecting D-Ser mitigates neuronal loss in the medial entorhinal area (MEA), whereas neuronal loss is the hallmark of TLE [43]. In addition, neuroinflammation was alleviated by D-Ser via reduction of astrocyte counts in the MEA, alerting the reactive status, and mitigating proliferation or infiltration of microglia to MEA [43]. Intense research revealed that the action of D-Ser in MEA may, through the temporoammonic pathway, alter the pathology of TLE in hippocampus [42]. 

In the model of rats with seizures induced by kainic acid, preload of L-Leu protects mice against seizure [44], but D-Leu has better effects than L-Leu or other anti-seizure medications. It potently terminates even ongoing seizures, elevates seizure threshold in mice, and attenuates long-term potentiation (LTP) in the hippocampal CA1 region but not basal synaptic activity [44]. Whereas other medications completely inhibit LTP, the inhibition of LTP may have a deleterious effect on learning and memory [44]. 

### 2.3. Improving Emotion, Motot, Preference, Memory, and Cognitive Ability

The function of NMDAR is important for learning, memory, etc., and memory and cognitive ability can thereby be improved by enhancing NMDAR-mediated neurotransmission. Administering D-Ser can improve spatial memory, learning, problem solving, depression or anxiety, and cognitive deficits, etc. in humans or rats through activating NMDAR [34,45,46,47]. In addition, some new pathways have been proposed. For instance, the c-Jun N-terminal kinase (JNK) signaling pathway. The mode of cognitive and motor deficits of Aβ1-42 injection mice was improved by intraperitoneally injected D-Ser for 10 consecutive days [48]. It was found that D-Ser restrained the JNK signaling pathway and elevated the clearance rate of Aβ [48]. D-Ser can attenuate toxicity damage to neurons as well as memory impairment in rats, which suffered from chronic lead exposure [49]. Particularly, D-Ser effectively improved the expression levels of NMDAR subtype NR2A [49]. In addition, D-Ser produces anxiolytic and precognitive effects in rats through intraperitoneal injection [50]. Inhibiting the signaling pathway of nucleus accumbens (NAc) brain derived neurotrophic factor (BDNF) has been put forward as a therapeutic approach for depression [51,52]. Particularly, injecting D-Ser into NAc suppresses depression through down-regulating the BDNF signaling pathway and regulating synaptic plasticity of NAc [51]. D-Ala can effectively inhibit hyperactivity in rats induced by methamphetamine, which is related to the reduction of excitatory amino acidergic neurotransmission [53]. Interestingly, D-AAs influence diet. Intraperitoneal injection or oral administration of D-Ser inhibits high-fat diet (HFD) intake and influences preference in male mice through regulating NMDAR [54,55].

**Figure 3:**
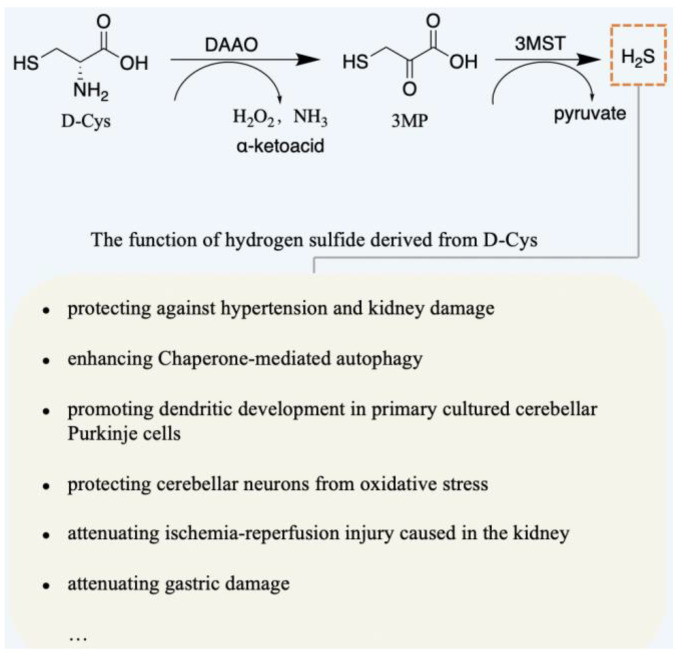
H_2_S is generated from D-Cys via two processes, oxidation of DAAO and catalysis of 3MST. During oxidation, some D-AAs are eventually converted into H_2_O_2_, NH_3_, and α-ketoacid. Apart from H_2_S, pyruvate is another metallic form of 3MP. Some functions of D-Cys are associated with the generation of H_2_S.

Recently, D-Cys has been proven as an endogenous amino acid in mammals [56]. The functions of D-Cys are highly related to H_2_S [57,58]. H_2_S is a regulator of physiological functions, and it is involved in many physiological functions, including as a signaling molecule in the nervous system, regulating inflammation process, and improving the dendritic development of purkinje cells (PCs), etc. [59,60,61,62,63,64]. One of the pathways of production of H_2_S was induced by DAAO, which utilizes D-Cys to produce 3-mercaptopyruvate (3MP), followed by providing 3MP to 3-mercaptopyruvate sulfurtransferase (3MST) [58,65,66], as shown in Figure 3. Hence, the generation of H_2_S can be derived from D-Cys, and D-Cys significantly promotes the dendritic development of PCs also reflecting this [64,66,67]. The clinical hallmark of spinocerebellar ataxia (SCA) is loss of balance and coordination accompanied by slurred speech [68]. The motor hypofunction of SCA1 model mice can be ameliorated by intraperitoneally injecting D-Cys. Seki et al. found that D-Ser can ameliorate pathological process, including inhibiting the degeneration of cerebellar PCs, improving the expression of mGluR1, and inhibiting glial activation, etc. [67]. 

### 2.4. Acoustic and Visual Protection

The mechanisms of hearing loss resulted by noise exposure can be classified into two sides: direct mechanical trauma to the organ of corti and the metabolic stress associated with increased oxidative metabolism in the inner ear [69]. The latter indirectly damages tissue through reactive oxygen species (ROS) and reactive nitrogen species (RNS) [69,70]. Some cochlear pathologies confirmed the levels of reactive oxygen species (ROS) were increased by noise exposure. Further, it plays a crucial role in noise-induced hair cell death or damage [71,72]. The symptoms are associated with threshold shift and hair cell loss [69]. 

**Figure 4:**
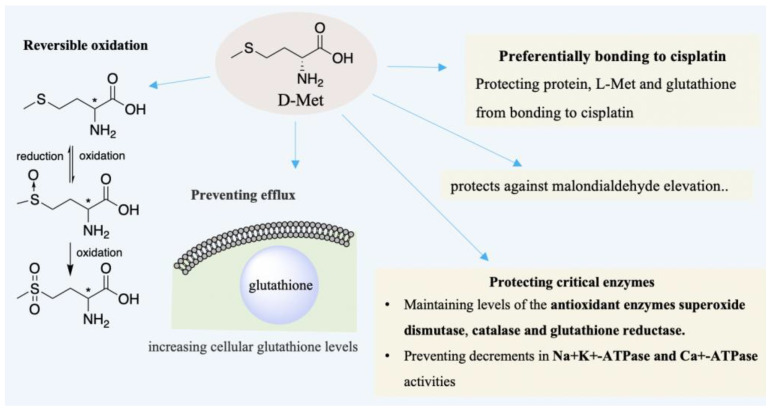
D-Met can be an otoprotective agent through its direct and indirect anti-oxidative action. It can be a free radical scavenger due to its reversible oxidation. Protecting critical enzymes, bonding to cisplatin, preventing the efflux of glutathione, and increasing cellular levels, etc., which are critical elements for indirect anti-oxidative function.

As early as in 1996, Campbell et al. had corroborated that D-Met can treat hearing loss induced by cisplatin (CDDP) [73]. In the case of cisplatin-ototoxicity, the protection provided by D-Met outperforms glutathione, glutathione ester, etc. [74]. Subsequently, Campbell, Schacht et al. found that D-Met is also effective for hearing loss induced by aminoglycoside and noise, respectively [74,75]. D-Met can attenuate oxidative stress to avoid hearing loss and sensory cell death [70], as well as alleviating loss of inner or outer hair cells [73,76]. D-Met has been proven as an effective otoprotective agent and successfully applied in many varied models [70,77,78,79,80]. In noise-induced hearing loss (NIHL), D-Met protects hearing loss and sensory cell death from oxidative stress generated by excessive noise exposure [70]. The otoprotective ability of D-Met may be based on its direct and indirect antioxidant action [70,74]. As shown in Figure 4, D-Met can be a free radical scavenger due to its reversible oxidation [81]. In addition, the indirect antioxidant action can also be derived from chirality selection that CDDP may preferentially bind to free D-Met to protect protein or L-Met from CDDP [73]. Additionally, it prevents efflux of glutathione from cells, whereas glutathione could be a cellular antioxidant to remedy the cell damage by ROS [69,70]. Further, the antioxidant function of D-Met may also be through protecting critical enzymes [74]. Supplementing D-Met elevates the levels of cellular glutathione and maintains the levels of the antioxidant enzymes superoxide dismutase, catalase, etc. [70,74,82]. Moreover, D-Met mitigates the decrease of Na+K+-ATPase and Ca+-ATPase activities [74,83,84]. 

The NMDA subtype of glutamate receptor and D-Ser play important roles in the visual cortex and retina [85]. D-Ser improves synaptic plasticity as well as the deficient function of NMDAR [85]. In particular, it was found D-Ser enhances visual cortical plasticity in adult mice [86]. Staubli et al. confirmed that increasing extracellular D-Ser levels is an effective strategy for remedying visual loss induced by malfunction of retinal cells [85]. Furthermore, D-Ser enhances the light-evoked responses in retinal ganglion cells, and it may regulate light-evoked synaptic responses through activating NMDAR [85,87,88].

### 2.5. The Analgesic Effect and Protective Action of D-AAs on Nervous System

A reduction in other neurological symptoms, such as analgesia, was observed in the D-AAs treatment. The earlier findings revealed that excitatory amino acid analogs have an antinociceptive effect in rats through administration in periaquedactal gray [89]. Activating NMDAR is also important for the analgesic effect of D-AAs. D-Ser has an antinociceptive effect on both acute and chronic pain induced by formalin via activating NMDAR [90]. It is also effective in the treatment of trigeminovascular headache in the rat model [91]. Yoshikawa et al. demonstrated that intracerebroventricular administration of D-Ser produces the antinociceptive effect in tail-flick by activating the supraspinal NMDAR [92]. D-Ser can enhance the antinociceptive effect of morphine [92]. Moreover, the selective antagonist of the glycine site of the NMDAR L-701,324 suppresses the antinociceptive effect of D-Ser or morphine [92]. Therefore, the activation of NMDAR may be crucial to the opioid receptor-mediated antinociception [93]. Notably, morphine promotes the mRNAs expression of the serine racemase and DAAO in rat brain, implying the close connection between NMDAR and the opioid receptor-mediated antinociception [93], and naloxone (a nonselective opioid receptor antagonist) mitigates the antinociceptive effect of D-Ser also reflecting the above connection [92]. D-Asp can mitigate the mechanical allodynia and ameliorate sensorial chronic pain through glutamate neurotransmission normalization in neuropathic mice [94]. Several variations were observed, including extracellular D-Asp were recovered to physiological levels and Shank1 and PSD-95 protein levels in the medial prefrontal cortex (mPFC) were reduced, etc. [94]. Moreover, high levels of D-Asp could reduce the nociceptive threshold in physiological and chronic pain conditions [95]. The mechanism is not fully known, but NMDAR is essential to the central sensitization at the spinal cord dorsal horn level [95]. In addition, D-Phe inhibits brain enkephalinase and provides analgesic help for acute pain or chronic intractable pain [96].

The protective effects of D-AAs on nerves tissue were also observed. D-Asp has a significant influence on the nervous system [97,98]. It alleviates the severity of experimental autoimmune encephalomyelitis [97], and it also alleviates the synaptic plasticity decay in the hippocampus of aged animals through enhancing the NMDAR-dependent long-term potentiation [99]. D-His has neuroprotective activity in the mode induced by Zinc [100]. As aforementioned, D-Met is an antioxidant. It protects cortical networks against neurotoxicity induced by cisplatin, as well as protecting plasmid DNA against damage induced by therapeutic carbon ions [101,102]. D-Cys protects cerebellar neurons from oxidative stress by generating H_2_S and scavenging ROS, etc. [58,61,65]

## 3. D-AAs Protect Tissue/Organ against Injury

### 3.1. Acute Kidney Injury (AKI)

Takashi Wada et al. corroborated that D-Ser and D-Ala have protective effects on AKI induced by ischemia-reperfusion (I/R) injury in the kidney [103,104]. In the case of D-Ser, the results suggested D-Ser is instrumental in ameliorating AKI that it suppresses the damage and promotes the hypoxia-mediated proliferation of tubular epithelial cells [103]. The symptoms of tubular injury were lessened in the germ-free mice after getting fecal transplantation from normal mice or oral administrate D-Ser [103]. It implies that gut microbiota is also important to AKI, which can be the source of D-Ser [103]. D-Ala followed a different mechanism in that it maintains mitochondrial membrane potential and inhibits the production of ROS via regulating NMDAR signaling. These effectively alleviate tubular epithelial cell (TECs) necrosis, which suffered from hypoxic stimulation, and the proliferation of TECs was promoted by D-Ala [104]. Recently, the cellular proliferation and kidney enlargement controlled by D-Ser was thought to be via mammalian targeting of the rapamycin (mTOR)-related pathway [105]. 

D-Cys can also potently mitigate the I/R injury in kidney [58,65]. The mechanism is not well explored. It may be attributed to the generating H_2_S pathway, whereas H_2_S protecting the kidney from acute injury was well established [59,63]. Notably, D-Cys is superior to L-Cys on the protection of renal cortex [58]. 

### 3.2. Reproduction

ROS damages mitochondria, DNA, protein, and oocyte maturation apoptosis [106]. D-Leu can mitigate oocyte maturation failure and inhibit the increase of alanine aminotransferase levels induced by restraint stress [106]. The proposed mechanism is that D-Leu upregulates the expression of Heme-oxygenase-1 (HO-1) and superoxide dismutase2 (SOD2) in ovaries. It might be through the Keap1/Nrf2 pathway, whereas the transcription factor Nrf2 associated with the expression of HO-1 and several SODs [106]. 

D-Asp plays a crucial role in regulating reproductive activity, especially controlling the synthesis and secretion of several hormones in mammalian endocrine systems [98,107,108]. Supplementing D-Asp improves spermatogenesis and sperm motility [109,110]. It is associated with several pathways, including the hypothalamic–pituitary–gonadal axis [111], upregulating the expression of prolyl endopeptidase (PREP) [112], increasing the concentration of extracellular Ca^2+^ mediated by NMDAR, generating specific sex hormone binding protein via stimulating Leydig cells, and directly affecting spermatogonial mitotic activity, etc. [108,109,113,114]. Accumulating investigations suggest that it is a rather complex process.

### 3.3. Hypertension, Gastric, Colitis

D-Cys prevents hypertension induced by high-salt in rats through regulating oxidative stress and generating H_2_S, etc. [57]. D-Cys could indirectly regulate the renin-angiotensin system through downregulating the concentration of renal angiotensin I/II and upregulating the protein levels of the angiotensin II receptor, etc. [57]. In addition, D-Cys indirectly protects mice from gastric damage via generating H_2_S [66]. D-Arg has superoxide anion O_2_^−^-scavenging potential in vitro [115]. However, the antioxidant activity of D-Arg in vivo is rather weak, but D-Arg can slightly improve endothelium-dependent relaxation in aorta ex vivo and partly alleviate the rise in systemic arterial pressure in rats [116]. D-Ser inhibits the development of chronic colitis induced by the adoptive transfer of naive T cells in recombination activating genes (RAG) deficient mice [117]. It can directly act on T cells and prevent T cell infiltration into the lamina propria and crypt elongation. Moreover, D-Ser reduces the amount of CD4+ T cells in the spleen as well as suppresses CD4 T cell proliferation and differentiation into Th1 and Th17 cells [117]. 

D-Trp can decrease the production of T_H_2 cytokines and chemokines, as well as restructuring a healthy microbial community genotype [118]. Nathan et al. confirmed that D-Trp directly intervenes in immune homeostasis, or indirectly by changing the microbiome of host [118]. It is therefore an immunomodulatory substance [118]. Supplementing D-Trp ameliorates allergic airway inflammation and hyper-responsiveness in mice [118]. In addition, the metabolism of D-AAs in the intestine produces H_2_O_2_, inhibiting some intestinal pathogenic bacterial infections [119].

## 4. D-AAs Protect Normal Cell from Radiation or Chemotherapy and Inhibits the Growth of Tumor Cell or Contamination Cell

Accumulating investigations of co-culture in vitro suggested D-AA has a deteriorative effect on cancer cells. For instance, while the breast cancer cell MCF-7 incubated in D-Leu solution (50 mM) for 24 h, the growth of MCF-7 cells was inhibited [120]. D-Met prolongates the survival time of AH109A hepatoma-bearing rats as it maintains the levels of transferrin and albumin in plasma [121]. Another similar investigation confirmed that the tumor growth, and synthesis of protein in tumor cells were inhibited by D-Met [122]. Furthermore, a comparative study where D-Met, D-Val, D-Leu, and D-Phe were allowed to treat AH109A hepatoma-bearing rats suggested that they were effective in inhibiting tumor cell except for D-Phe [123]. Among these, the efficiency of D-Val was significantly higher than others [123]. DNA, RNA, and protein content in the tumor tissues and tumor volume, etc. were reduced [123]. The levels of hematocrit and hemoglobin red blood cell were higher than the control groups, suggesting that D-AAs solution also improves the nutritional status of the host [123]. Further, D-Val can reduce the contamination of fibroblast in the co-culture of endometrial stromal cells and human myometrial cells by selectively inhibiting fibroblast proliferation [124,125].

Aside from hearing protection, D-Met also prevents mucosal tissue from injury induced by radiation or chemotherapy [126,127]. Campbell et al. confirmed that D-Met can be a cytoprotectant in CDDP treatment; it effectively protects the stria vascularis against several types of induced damage, as well as reducing the death rate in the ovarian cancer treated [128,129]. Recently, a study revealed D-Met protects orals mucosa from radiation-induced cytotoxicity, by providing selective protection on nontransformed human cells and suppressing the burst of ROS in nontransformed human cells, etc. [127]. However, the controlled experiment suggested D-Met was a rather weak free-radical scavenger [127]. Further studies are necessary to elucidate the mechanisms of D-Met protection. Overall, D-AAs showed tremendous success in treating varied diseases or symptoms. Table 1 is a summary with respect to some representative experiments.

## 5. D-AAs Inhibit the Formation of Biofilm and Enhance Antibiotic Effect 

Wound infection, dental caries, periodontitis, and otitis media are generally induced by biofilm bacteria [130]. Biofilm is frequently encountered in natural microbial biomes and structurally complex, dynamic systems [131]. It is generated from bacterial cells approaching and depositing on the surfaces, then proliferating and embedding in the mixture of protein exopolymers, exopolysaccharides (EPS), extracellular DNA, etc. to eventually form consecutive biofilm [132,133]. Biofilm protects cells from hostile environmental insults, antimicrobial agents, etc. [130,131]. Further, it assists microbes to produce wound infection, giving rise to inflammation [134,135]. D-AAs are potent agents to disperse biofilm and inhibit the formation of biofilm [2,136]. Richard Losick et al. confirmed that TasA fibers were disengaged from their cell wall while the terminal D-Ala of the peptide side chains of peptidoglycan was replaced by other D-AAs [8,136]. Further investigation revealed that misincorporation into protein may account for the biofilm disengaging effects of D-AAs [137]. In addition, some investigations found that D-Ser inhibits the expressions of the genes with regard to the attachment and formation of biofilm, such as AgrA, SarS, IcaA, etc. [138], however, it is not fully known and further studies are necessary to elucidate the mechanism.

Several reports have established the dissociation of biofilm by D-AAs. D-Leu, D- Met, D-Tyr, D-Trp, D-Cys, D-Ser, D-Val, and D-Phe are effective in inhibiting biofilms formed by varied bacteria, including *Bacillus subtilis*, *Streptococcus mutans*, and *Streptococcus sanguinis*, etc. [136,138,139,140,141,142,143]. However, mixtures of D-AAs have suggested a stronger effect on biofilm [144,145]. For instance, the mixture of D-Met, D-Phe, and D-Trp was more effective in dispersing biofilms of *Staphylococcus aureus* and *Pseudomonas aeruginosa* than singular D-AA [144]. In particular, while D-AAs concurrently work with antibiotics, the antibacterial effects of antimicrobial agents would be enhanced [144,145]. Relevant animal experiments are rare. Takashi Wada et al. showed that D-Ser reduced catheter infection of methicillin-resistant *Staphylococcus aureus* in the model of murine peritonitis [138]. More relevant animal experiments may occur in the near future.

## 6. Incorporating D-AAs into Drugs Afford Stronger Biostability and Efficiency

**Figure 5:**
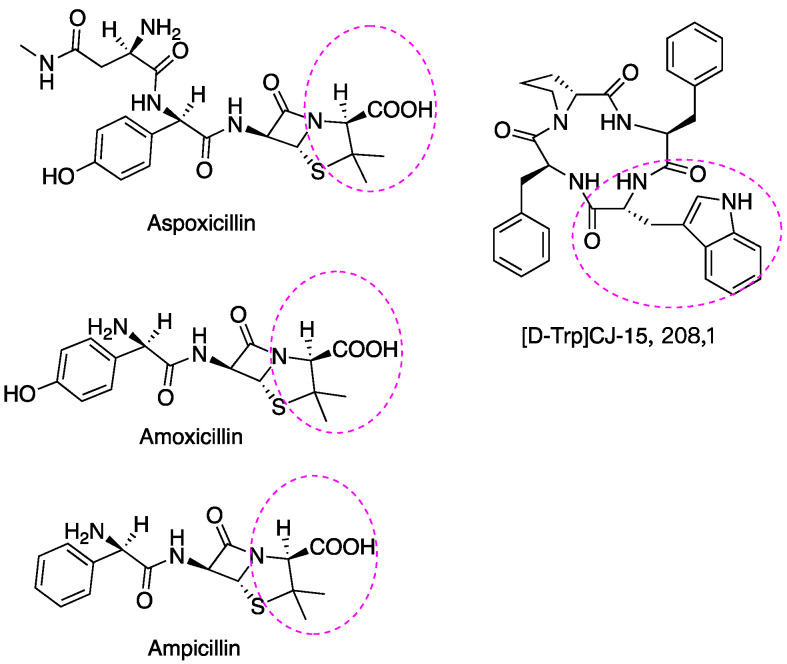
D-Val and D-Trp are incorporated into some antibiotics and analgesics drug, respectively.

**Figure 6:**
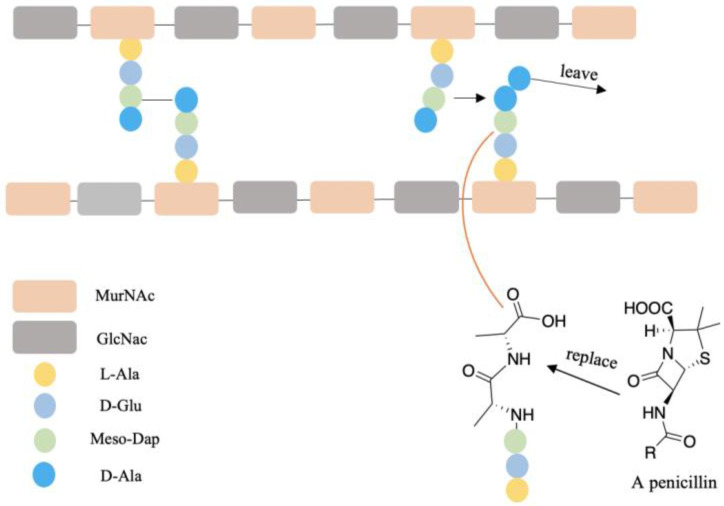
The possible antibiotic mechanism of penicillin. The peptidoglycan contains temporary D-alanyl-D-ala groups during crosslinking processes. Crosslink would be terminated while D-alanyl-D-ala was replaced by penicillin. The glycan strands consist of alternating N-acetylglucosamine (GlcNAc) and N-acetylmuramic acid (MurNAc). The crosslinking of glycan strands is through the connection of meso-diaminopimelic acid (meso-Dap) and D-Ala.

A few examples of antibiotic drugs containing D-AAs include: aspoxicillin, amoxicillin, and ampicillin, which contain D-Val and are shown in Figure 5. (D-Trp)CJ-15,208 has an analgesic effect [146]. The biostability of peptides was improved by incorporating D-AAs into its structure because D-AAs containing peptides are resistant to enzymatic degradation [147,148,149]. Hence it has a longer half-time in vivo circulation, leading to the drug delivery system being more efficient [150]. Moreover, the introduction of D-AAs in peptides could increase receptor binding affinities [151]. The action mechanism of penicillin and its analogs may be through replacing the D-Ala-D-Ala motif of peptidoglycan since these have similar structures (Figure 6) and the normal synthetic processes would be interrupted [152,153]. At present, D-peptide is frequently used, and it is recognized as a potent and promising drug. Enkephalin (D-Ala2, D-Leu5) is effective to attenuate cerebral I/R injury [154]. D-Trp-6-LH-RH is used for the treatment of advanced prostatic cancer/advanced ovarian cancer, etc. [155,156]. Furthermore, D-AAs derived positron emission tomography (PET) radiotracers have demonstrated promising application in clinical diagnosis, including brain tumor, brain glioma tissue, and bacterial infection, etc. [157,158,159]. It has better specificity and absorption in tumor/infected tissue, as well as outperforming its L enantiomers [157,158,159].

## 7. The Source and Administration of D-AAs for Trials Using, as well as Safety

As shown in Table 1, oral administration is the only approach in clinical trials. Intraperitoneal injection, or intravenous infusion, etc. are prevalent among animals, and they appear to have a higher risk in clinical trials. However, targeted administration is an effective approach, especially when the D-AAs derived from oral administration were digested by gut microbiota. Most D-AAs are commercially available with a purity over 99%. D-AAs are derived from industrial production, including chemical synthesis, fermentation, and enzymatic transformation [160]. Purification and separation are essential for most protocols. Compared to conventional methods, enzymatic transformation is a promising approach, which can yield D-AAs with high optical purity and productivity and generate less pollution [160].

Although growing evidence suggests D-AA has a therapeutic or protective effect in the vivo of mammals, the toxicity and side effects cannot be ignored. Some D-AAs have deleterious effects. For instance, high dose excitatory AA can lead to excitotoxicity, which is a persistent or excessive stimulation of their receptors [161], and the overdose of D-Ser results in misalignment of muscle fibers and motor neuron defects [162]. At present, the known side effects are rare since the number of relevant clinical trials is not significant and existing clinical trials appear to have not gone beyond phase II (Table 1). Moreover, most experiments dwell on the animal stage (Table 1). According to human trials, at present, the effect of D-AAs is acceptable. Further phase studies are necessary to thoroughly confirm the safety of D-AAs, and there is a tendency that growing numbers of clinical trials would be disclosed.

## 8. Conclusions

Numerous investigations have confirmed the trials relating to the D-AAs treatment in humans or animals and that it improves symptoms through oral administration or injection. D-AAs have more prominent advantages compared to other therapeutic agents in that it exists in biofluids where mainly derived from food uptake or gut microbiota generating, and particularly, it can regulate the function of organisms. Therefore, administration of D-AAs may have a better effect and need less effort to evaluate side effects or toxicities. In addition, incorporating D-AAs into drugs, including antibiotics, might have great prospects. Overall, the clinical application of D-AAs will be more extensive in the near future.

## Figures and Tables

**Table 1:** Representative experiments of varied D-AAs with regard to different diseases/symptoms.

D-AA	Subject	Diseases/Symptoms	Method	Period	Side Effects	Reference
D-Ala	Human	Schizophrenia	Oral administration, D-Ala 100 mg/kg daily (mixed with orange juice)	6 weeks	Insomnia, nausea (short-lived and resolved spontaneously)	[36]
D-Met	male Chinchillas laniger	Hearing loss induced by noise	Intraperitoneal injection, D-Met saline 200 mg/kg (12 h intervals) after noise exposure	2 days	-	[70]
D-Met	Male Wistar rats	Hearing loss induced by cisplatin	Intraperitoneal injection, D-Met saline at 300 mg/kg	Once (prior to the infusion of CDDP)	-	[73]
D-Met	Pigmented male guinea pigs	Hearing loss induced by aminoglycosides	Intraperitoneal injection, D-Met saline twice daily at 200 mg/kg each	6.5 weeks	-	[75]
D-Met	Pigmented guinea pigs	Kanamycin-induced ototoxicity	Intraperitoneal injection, D-met (120, 180, 240, 300, 360, 420, or 480 mg/kg/day) twice per day	23 days	-	[77]
D-Met	Male donryu rats	The survival time of tumor-bearing rats	Intravenous infusion, amino acid mixture 230/kg/day (D-Met, 0.35 g/mL)	7 days	-	[121]
D-Met	Human	Mucositis induced by radiation and chemotherapy	Oral administration, D-Met 100 mg/kg b.i.d.	Twice (before and after radiotherapy)	Nausea, vomiting	[126]
D-Ser	Human	Schizophrenia	Oral administration, D-Ser 30 mg/kg daily (mixed with orange juice)	6 weeks	Insomnia, nausea, diarrhea, constipation (short-lived and resolved spontaneously)	[19]
D-Ser	Human	Post-traumatic stress disorder	Oral administration, D-Ser 30 mg/kg daily	6 weeks	No significant side-effects	[40]
D-Ser	Human	Parkinson’s disease	Oral administration, D-Ser 30 mg/kg daily	6 weeks	No significant side-effects	[41]
D-Ser	Adult rats	Temporal Lobe Epilepsy	Cannulation surgery, continuous infusion of D-Ser (100 μM at 0.1 μL/h)	29 days	-	[43]
D-Ser	Older adults	Improving spatial memory, learning and problem solving	Oral administration, D-Ser 30 mg/kg daily (mixed with orange juice)	Approximately 3 months	-	[45]
D-Ser	Adult male mice	Cognitive deficits	Intraperitoneal injection, D-Ser saline (1 g/kg or 100 mg/kg)	Once	-	[46]
D-Ser	male Sprague-Dawley rats	Memory impairment and neuronal damage induced by chronic lead exposure	Intraperitoneal injection, D-Ser 30 and 60 mg/kg, twice a day	8 weeks	-	[49]
D-Ser	gustatory cortex rats and Wistar rats	Anxiety-like Behavior and Spatial Learning Ability	Intraperitoneal injection, D-Ser 50 mg/kg or 100 mg/kg	Once	-	[50]
D-Ser	Adult male mice	Depression	Intranucleus accumbens infusions, D-Ser (2 µg/perside, 5 µg/perside)	14 days	-	[51]
D-Ser	Male mice	High-fat diet intake and preference	Intraperitoneal injection, D-Ser 1, 2, or 4 g/kg body weight	Once	-	[54]
D-Ser	Rabbits and rats	Retinal dysfunction	Intraperitoneal injection, D-Ser 600 mg/kg; intravitreal injection D-Ser 12 μmol	Once	-	[85]
D-Ser	Male Wistar rats	Antinociception	Intracerebroventricular administration, dose-dependent experiments, D-Ser (10–40,000 nmol); In combination studies, D-serine (10 μmol)	Once	-	[92]
D-Ser	Mice	Acute kidney injury	Oral administration D-Ser 20 mM or fecal microbiota transplantation	-	-	[103]
D-Ser	Wild-type mice	Colitis	Oral administration, 1.5% (wt/vol) D-Ser diluted into H2O	7 days	-	[117]
D-Cys	Spontaneously Hypertensive Rats	Hypertension and Kidney Damage	Gastric intubation, D-Cys (8 mmol/kg body weight/day) between 4 and 6 weeks of ages	14 days	-	[57]
D-Cys	Mice	Kidney injury from ischaemia-reperfusion	Gastric intubation, 8 mmol/ kg body weight	Once	-	[58]
D-Cys	Mice	Gastric damage	Oral administration, D-Cys 100 mg/kg	Once	-	[66]
D-Cys	Mice	Spinocerebellar ataxia	Intraperitoneal injection, D-Cys 100 mg/kg	10 weeks	-	[67]
D-Leu	Male mice	Seizure	Intraperitoneal injection, D-Leu 300 mg/kg after kainic acid injected	Once	-	[44]
D-Leu	Female mice	Oocyte maturation failure	Oral administration, commercial powder diet containing 0.3% d-Leu	14 days	-	[106]
D-Leu	Male Donryu rats	The survival time of tumor-bearing rats	Intravenous infusion, amino acid mixture 230/kg/day (D-Leu, 1.25 g/mL)	7 days	-	[123]
D-Asp	Spared nerve injury mice	Pain and cognitive impairment	Oral administrate, D-Asp water solution 20 mM	30 days	-	[94]
D-Asp	Male mice	Hippocampal age-related synaptic plasticity deterioration	Oral administrate, D-Asp 20 water solution mM	3 months or 12 months	-	[99]
D-Asp	Sexually mature New Zealand rabbit males	Sperm quality	Oral administration, DL-Asp mixed with food, 1.3 g DL-Asp/head	14 days	-	[109]
D-Arg	Male Sprague-Dawley rats	Blood pressure	Daily subcutaneous injection of 3 mL d-Arg (1 M)	7 days	-	[116]
D-Trp	Female mice	Allergic airway disease	Oral administration, D-Trp 0.9 mg/day per mouse	3 days	-	[118]
D-Val	Male Donryu rats	The survival time of tumor-bearing rats	Intravenous infusion, amino acid mixture 230/kg/day (D-Val, 0.45 g/mL)	7 days	-	[123]

- Not mentioned or unclear in the original text.
